# Network Analysis Identifies *ELF3* as a QTL for the Shade Avoidance Response in Arabidopsis

**DOI:** 10.1371/journal.pgen.1001100

**Published:** 2010-09-09

**Authors:** José M. Jiménez-Gómez, Andreah D. Wallace, Julin N. Maloof

**Affiliations:** Department of Plant Biology, College of Biological Sciences, University of California Davis, Davis, California, United States of America; University of Georgia, United States of America

## Abstract

Quantitative Trait Loci (QTL) analyses in immortal populations are a powerful method for exploring the genetic mechanisms that control interactions of organisms with their environment. However, QTL analyses frequently do not culminate in the identification of a causal gene due to the large chromosomal regions often underlying QTLs. A reasonable approach to inform the process of causal gene identification is to incorporate additional genome-wide information, which is becoming increasingly accessible. In this work, we perform QTL analysis of the shade avoidance response in the Bayreuth-0 (Bay-0, CS954) x Shahdara (Sha, CS929) recombinant inbred line population of Arabidopsis. We take advantage of the complex pleiotropic nature of this trait to perform network analysis using co-expression, eQTL and functional classification from publicly available datasets to help us find good candidate genes for our strongest QTL, *SAR2*. This novel network analysis detected *EARLY FLOWERING 3* (*ELF3;* AT2G25930) as the most likely candidate gene affecting the shade avoidance response in our population. Further genetic and transgenic experiments confirmed *ELF3* as the causative gene for *SAR2*. The Bay-0 and Sha alleles of *ELF3* differentially regulate developmental time and circadian clock period length in Arabidopsis, and the extent of this regulation is dependent on the light environment. This is the first time that *ELF3* has been implicated in the shade avoidance response and that different natural alleles of this gene are shown to have phenotypic effects. In summary, we show that development of networks to inform candidate gene identification for QTLs is a promising technique that can significantly accelerate the process of QTL cloning.

## Author Summary

A major interest in evolutionary biology is to understand the genetic mechanisms that underlie phenotypic variation in nature and how they interact with the environment. A good example of adaptive genetic variation in response to the environment is the shade avoidance response. Although some plant groups try to outgrow their competitors when shade cues are detected others do not, as they are adapted to live in constitutive shade, such as a forest canopy. We used a segregating population derived from two Arabidopsis ecotypes to investigate this variation and found a chromosomal region affecting the shade avoidance response. We developed a network analysis method that combines genomic information from publicly available databases to identify the causative gene in that interval as *ELF3*. Using genetic and transgenic methods we confirmed the effect of *ELF3* in the shade avoidance response, and showed that different alleles of this gene in natural populations of Arabidopsis result in different developmental times and circadian periodicity depending on the environmental conditions.

## Introduction

As sessile organisms, plants exhibit an extraordinary phenotypic plasticity that allows them to optimize their development and metabolism according to environmental cues. Among the signals that plants perceive and respond to, light plays a fundamental role as a source of information and energy. A typical example of light as an information source that modulates plant behavior is the shade avoidance response [1]. Because plant tissues absorb red light and reflect far red light, plants can detect the proximity of neighbors by a decrease in the red to far-red ratio (R:FR) and trigger the shade avoidance response [2].

The molecular changes induced by shade avoidance are diverse and complex at all levels. Foliar shade is mainly perceived by the red and far-red phytochrome photoreceptors *PHYTOCHROMES D* and *E* (*PHYD* and *PHYE*; AT4G16250 and AT4G18130), and especially *PHYTOCHROME B* (*PHYB*; AT2G18790), but general decreases in intensity are sensed by the blue light photoreceptors, cryptochromes and phototropins, and can be important in response to shade [1], [3], [4]. Phytochromes initiate the response through interaction with the phytochrome interacting factors *PHYTOCHROME INTERACTING FACTOR 4* and *5* (*PIF4* and *PIF5*; AT2G43010 and AT3G59060), and other transcription factors such as *PHYTOCHROME INTERACTING FACTOR 3-LIKE 1* (*PIL1*; AT2G46970) and *ARABIDOPSIS THALIANA HOMEOBOX PROTEIN 2* (*ATHB2*; AT4G16780) [5]–[7]. The complexity of the downstream signaling cascade is a consequence of activation of negative feedback loops on these transcription factors [8], [9], activation and cross-talk between multiple hormone signaling pathways [10], [11], and interactions with the circadian clock [6] among many other molecular processes [9].

Shade avoidance phenotypes include increased elongation of the hypocotyl and delay of cotyledon opening in seedlings, and increased elongation of stems and petioles, increased apical dominance and reduced developmental times in adults [1]. Although in seedlings the shade avoidance response is necessary to allow optimal positioning and immediate access to light, in adult crop plants this response is considered detrimental due to reduced plant biomass and fruit yield. Furthermore, the shade avoidance response can have adaptive value, conferring an advantage to plants in competitive environments but being maladaptive for plants growing in constitutive shade (i.e. under a forest canopy) [12]. Consistent with this idea, the degree of phenotypic plasticity to shade varies in natural populations depending on the light environment [12].

QTL analyses in immortal plant populations allow comparison of genetically identical individuals in different environmental conditions [13]. QTL analyses of the shade avoidance response have been carried out using seedlings from immortal populations of Arabidopsis grown under sun versus simulated foliar shade or red versus far-red light conditions [14]–[17]. In one of these studies, the blue light photoreceptor *CRYPTOCHROME 2* (*CRY2*; AT1G04400) was identified as the main cause of the differences in cotyledon opening in response to FR light between the Ler and Cvi accessions [17]. However, genetic sources of natural variation in the shade avoidance response in adult plants remain unknown, in part due to the low resolution typical of QTL mapping experiments [14].

A common challenge in QTL analysis is identification of the causal gene(s) among typically hundreds of candidate genes in the QTL confidence interval. Recombination-based fine mapping is still the most common method to identify causative genes. However, genomic resources in Arabidopsis such as genome sequences for several accessions [18], detailed genome annotations [19] and genome-wide expression profiling for innumerable conditions and genotypes [20], [21] provide potentially useful tools to aid in QTL cloning. Worth mentioning are the available genome-wide expression profiles for full segregating populations, which allow expression-QTL (eQTL) analyses that can suggest chromosomal regions responsible for variation in expression levels across the population [22], [23]. Compilation of genomic resources to identify QTLs has become an active and promising area of research in model organisms [24]–[29]. When successful, these approaches have the advantage of saving a significant amount of time in the laborious process of cloning a QTL, and can suggest novel candidate genes not considered *a priori*. The highly pleiotropic nature of the shade avoidance response suggests that the genes that control this response modulate multiple pathways. Therefore, the use of systems and network approaches seems an appropriate strategy to identify causative genes for QTLs affecting the shade avoidance response.

In this work, we performed QTL analysis of the shade avoidance response in the Bay-0 and Sha RIL population of Arabidopsis. We combined classical mapping and a novel network analysis to identify *ELF3* as a gene that differentially regulates the response in the population. *ELF3* is a gene of unknown molecular function that plays an important role in the plant circadian clock. This work implicates *ELF3* in the shade avoidance response for the first time and describes links between the circadian clock and the shade avoidance response.

## Results

### Mapping QTL for shade avoidance response in the Bay-0 x Shahdara RILs

Although the shade avoidance response has typically been studied in seedlings, its effect is observable throughout the life cycle of plants. To investigate natural variation in the shade avoidance response of adult Arabidopsis plants, we grew the Bay-0 x Sha RIL population under simulated sun and shade conditions in 12 hours light, 12 hours dark (12∶12) photoperiods. All plants were phenotyped for shade avoidance using plant size and morphology traits (leaf blade width and length, petiole length and leaf angle) and developmental time traits (bolting date, rosette diameter and leaf number at bolting, and flowering time). We calculated indices for each RIL, trait and condition using mixed effect models as detailed in Materials and Methods. In addition, for each RIL and trait we obtained two shade avoidance response indices: the subtraction index, by subtracting the shade indices from the sun indices; and the residual index, by regressing the simulated shade indices on to those for simulated sun and then taking the negative residuals of the regression. Most QTL analysis methods assume that trait values fit a normal distribution. We log-transformed those traits that did not meet this assumption (rosette diameter, bolting date, leaf number and flowering time, p<0.01 by Shapiro-Wilks test, see Materials and Methods). All shade avoidance response traits fit a normal distribution after transformation (Shapiro-Wilks test p>0.05). Among the traits measured, only leaf angle did not show a significant response to shade in a mixed effect model. Leaf blade length and width showed shade responsiveness, but did not present significant variation between RILs and were removed from the analysis (Figure S1). All other traits presented significant genotype x environment interactions (Figure S2).

**Figure 1:**
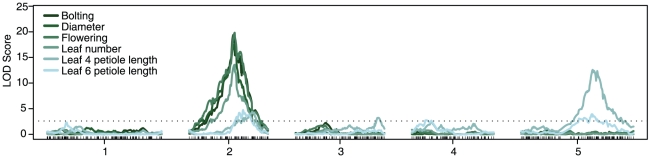
*SAR2* is the major QTL for the shade avoidance response. QTL analysis results for the shade avoidance response residual index in the Bay-0 x Sha recombinant inbred line population grown in 12:12 photoperiods. LOD score (y axis) from interval mapping results are plotted against all 5 Arabidopsis chromosomes (x axis). Tick marks on the x axis correspond to molecular markers in the genetic map of the Bay-0 and Sha RILs. A representative estimation of the LOD threshold is illustrated by a horizontal dotted line (average = 2.60, range = 2.48–2.75). *SAR2*, located on chromosome 2, is the largest effect QTL for the shade avoidance response.

QTL analysis was performed for all trait indices in simulated sun and simulated shade, and for the shade avoidance indices. We obtained similar results using the log transformed and untransformed data, and from the subtraction and residual shade avoidance indices (data not shown). Therefore, from now on we will only detail the results from the untransformed data and the residual shade avoidance response index. In general, QTL profiles from developmental time traits could be easily distinguished from those from plant size and morphology traits, suggesting different underlying genetic mechanisms (Figures 1 and S3). QTL profiles in the sun and shade environments were highly similar, indicating a common genetic control mechanism of these traits in both conditions (Figure S3). However, differences between the QTLs in both environments can be highlighted by the QTL analysis of the shade avoidance response index. In this analysis we detected a major QTL in chromosome 2 affecting all developmental time traits (*Shade Avoidance Response 2*, *SAR2*, Figure 1). Smaller effect QTLs for petiole length were located in chromosomes 2, 3 and 5 (Figure 1). For *SAR2*, the Bay-0 allele presented a greater shade avoidance response than the Sha allele by accelerating the development of shade-grown plants that carry this allele while having a reduced effect in sun-grown plants (Figure S4). *SAR2* explained between 27.6% and 30.4% of the variation in the shade avoidance response found in the population.

To characterize the effect of photoperiod in the shade avoidance response we measured developmental time traits in a subset of the RILs under 16 hours light, 8 hours dark (16∶8) photoperiods (see Materials and Methods). In this experiment we observed less variance of shade avoidance indices among the RILs than in the 12∶12 experiment (Figure S2). However, the positions and directions of the effects of the major QTLs in 16∶8, including *SAR2*, are in agreement with the results from the 12∶12 experiment (Figures 1, S3, S4 and S5). Interestingly, the number and positions of minor QTLs differ, suggesting the existence of small effect loci with photoperiod specificity. In general, reduced effects were found in the QTLs in 16∶8 photoperiods, which could be caused by the smaller number of lines assayed in 16∶8 or by reduced phenotypic differences caused by shorter developmental time in long days.

We decided to further investigate *SAR2*, the largest effect QTL affecting the shade avoidance response both in 12∶12 and 16∶8 photoperiods. For convenience, all following experiments were done in 16∶8 unless otherwise stated.

### QTL confirmation

**Figure 2:**
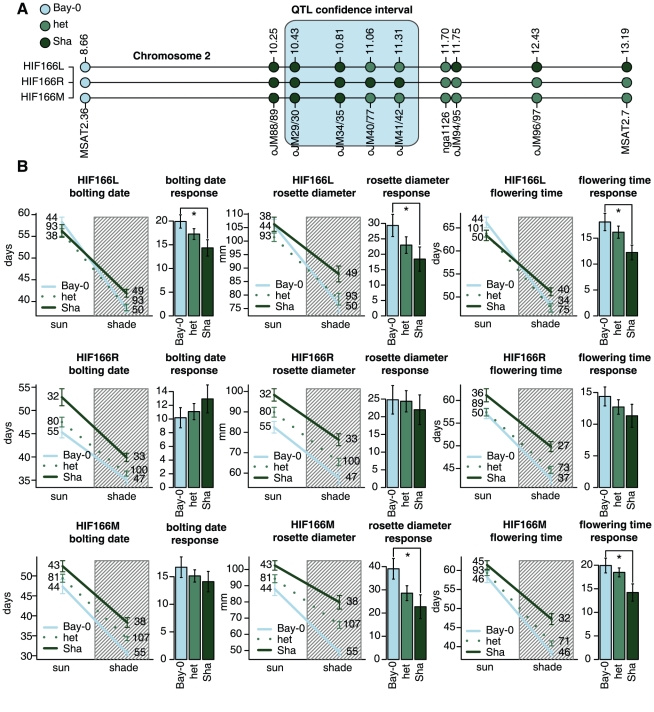
Confirmation of the shade avoidance response phenotypes and fine mapping of *SAR2* using HIFs. Fine mapping and phenotype characterization of heterogeneous inbred families segregating for *SAR2*. (A) Genetic map of *SAR2* region in chromosome 2. Circles represent molecular markers, with their corresponding names below and positions in megabases according to TAIR9 above (see Materials and Methods). The colored boxed area represents the 2-LOD confidence interval for *SAR2*. HIF166L, HIF166R and HIF166M are heterozygous for part of the confidence interval of *SAR2*, for a region downstream of *SAR2* or for both, respectively. (B) Phenotypes for the progeny of HIF166L, HIF166R and HIF166M. Bolting date, rosette diameter and flowering time were measured in the progeny of each HIF line both under simulated sun and shade conditions in 16∶8 photoperiods. After flowering, plants were assigned to a genotypic class using the molecular markers depicted in (A). Line plots represent averages ± standard errors for each genotype under each condition. Shaded and non-shaded areas in each plot indicate simulate shade and sun conditions respectively. Numbers indicate the plants used in the experiment. Bar plots to the right show the shade avoidance response index as the increase ± standard error of averages in simulated sun compared to simulated shade. Asterisks indicate significant differences between Bay and Sha alleles (ANOVA test p<0.05). HIF166L and HIF166M show significant shade avoidance response differences between alleles.

One way to characterize *SAR2* is to compare the shade avoidance response of plants inheriting the *SAR2* chromosomal region from either Bay-0 or Sha. We obtained two heterogeneous inbred families (HIFs) heterozygous for a region that included all (HIF144) or part (HIF166) of the *SAR2* confidence interval (Figure S6A). An initial screen of the progeny from both HIFs under simulated shade revealed differences in shade avoidance traits that correlated with the genotypes of the *SAR2* region, with Bay-0 alleles inducing shorter developmental times than Sha (Figure S6B). Since HIF166 was heterozygous for only part of *SAR2*'s confidence interval but still segregated for developmental time traits, we focused on this line to obtain recombinants that narrowed the position of the causative gene(s). Recombinant lines descended from HIF166 uncovered the existence of at least two loci on chromosome 2 controlling the studied traits. A first locus or group of loci is located within the confidence interval of *SAR2* and a second region is downstream and outside the confidence interval (Figure 2).

To investigate which one of these two regions was responsible for the shade avoidance response variation detected in our QTL analysis, we characterized the progeny of lines segregating for *SAR2* (HIF166L), the region distal of *SAR2* (HIF166R) or both regions (HIF166M) under simulated sun and shade conditions (Figure 2A). Progeny from HIF166L did not present differences attributable to the segregating alleles when grown in simulated sun but did under simulated shade (Figure 2B, line plots), resulting in significant differences in their responses to shade (Figure 2B, bar plots). The heterozygous progeny of HIF166L showed intermediate shade avoidance response phenotypes, suggesting a semi-dominant relationship between the Bay-0 and Sha alleles. The fact that Bay-0 alleles in the plants descended from HIF166L increased the response to shade in all traits is consistent with the QTL analysis and confirms the position of *SAR2* in the interval segregating in this HIF. On the other hand, the progeny of HIF166R showed differences in all traits measured both in simulated sun and shade, with the Bay-0 allele delaying flowering and increasing rosette diameter (Figure 2B, line plots). However, the similar magnitude of the effects under both light conditions implies that this downstream region is not significantly involved in the shade avoidance response (Figure 2B, bar plots). Corroborating these observations, the progeny of HIF166M, segregating both for *SAR2* and the downstream region, presented characteristics of both lines HIF166L and HIF166R: there were significant differences in the phenotypes measured in each treatment (Figure 2B, line plots), but those differences were larger under simulated shade treatment, resulting in differential shade avoidance response (Figure 2B, bar plots). It is worth mentioning that although plants descended from HIF166M presented differences in the shade avoidance response attributable to the segregating alleles as expected, these differences were not significant for bolting date measurements. This could be a secondary effect of the early bolting phenotypes caused by the region downstream of *SAR2* reducing the time that plants have to exhibit significant differences.

Altogether, these results suggest the existence of at least two loci affecting developmental time related traits on the studied interval in chromosome 2. The locus or loci outside of the QTL confidence interval has similar effects on these traits in all light conditions tested here, while the effect of *SAR2* depends on the light condition and therefore alters the shade avoidance response of the plants tested.

### Network analysis

**Figure 3:**
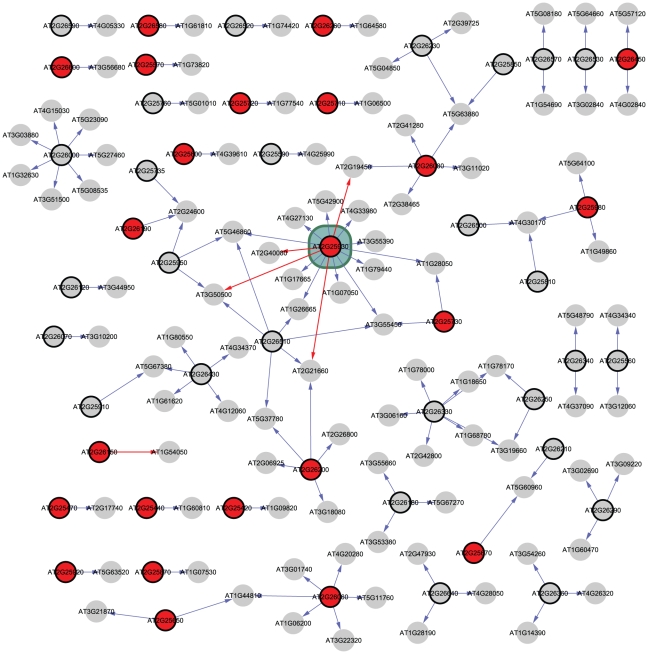
Network analysis identifies *ELF3* as a candidate for *SAR2*. Network analysis for the 133 genes located in the intersection of the *SAR2* QTL confidence interval and the HIF166L heterozygous interval. Nodes represent genes. Only nodes with at least one edge are represented. Nodes with thick borders are the 133 candidate genes located in the interval. Edges connect genes that are co-expressed with the candidate gene and have an eQTL in the position of the candidate gene. Edges colored in red connect genes that share one or more functional category. Red colored nodes represent genes with non-synonymous polymorphisms between Bay-0 and Sha. The node representing the *ELF3* gene, which has more connections to functionally related genes than any other node in the network, is enclosed in a colored box.

Together, the chromosomal region delimited by the *SAR2* confidence interval and the heterozygous region in HIF166L includes 363 genes, some of which are related to light signaling or response, such as several encoding F-box proteins, *ATTENUATED FAR-RED RESPONSE (AFR*; AT2G24540), *ELF3*, *CONSTANS-LIKE 3* (*COL3*; AT2G24790) and *ARABIDOPSIS RESPONSE REGULATOR 12* (*ARR12;* AT2G25180) among others [30]–[34]. To help us elucidate the causative gene for *SAR2*, we constructed networks for all 363 candidates. First, since genes in the same pathways or in the same functional complexes often exhibit similar expression patterns under diverse temporal and physiological conditions, we connected each candidate gene to co-expressed genes across 1388 microarray experiments [20]. Next, we expect causative genes underlying *SAR2* to modulate the genes in their networks to generate the shade avoidance phenotype. For this reason, we filtered the networks to keep only co-expressed genes with an eQTL in the location of the candidate gene, indicative of a regulatory relationship [35]. To avoid arbitrary associations between pairs of genes, we considered only those networks that had connections between genes with similar functional classifications as determined by having similar GO categories (see Materials and Methods) [19]. Finally, since we anticipate the causative gene for *SAR2* to be polymorphic between Bay-0 and Sha, we searched the candidate genes for non-synonymous polymorphisms or for the presence of cis-eQTLs indicative of promoter or auto-regulatory changes [18], [35]. Figure 3 shows the network result for the 133 genes found in the intersection of the QTL and HIF166L intervals. We obtained similar results for the combination of these intervals (Figure S7). Both network analyses indicated *ELF3* as the candidate gene with the highest evidence for differential control of expression of related genes in the Bay-0 and Sha population. In addition, according to re-sequencing datasets, *ELF3* contains amino acid substitutions between Bay-0 and Sha in a conserved domain of the protein [18], [36]. Further sequencing of the Bay-0 and Sha alleles of *ELF3* revealed the insertion in the Bay-0 allele of 8 glutamines in a tri-nucleotide repeat region that had been identified before as polymorphic among natural populations of Arabidopsis (Figure S8) [37]. Given the prominent placement of *ELF3* in the network analysis we concentrated on *ELF3* as a likely candidate for *SAR2*.

### Quantitative complementation test

**Figure 4:**
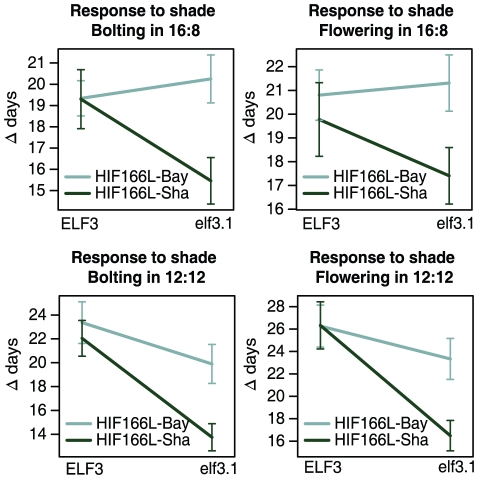
ELF3 affects development time in quantitative complementation test. Allelic complementation of ELF3 interaction with the environment. Quantitative complementation tests were performed by combining different alleles at *ELF3* in F1 backgrounds. The relative complementation of either *elf3-1* mutant allele or *ELF3-Col* (the Columbia wild type) by an *ELF3-Bay-0* or *ELF3-Sha* allele was measured through bolting date and flowering time both in 16∶8 and 12∶12 photoperiods. Each data point represents the difference of the means from plants in simulated sun minus the means from the plants in simulated shade. 31 to 56 individual plants were measured per genotype and environment in 16∶8, and 56 to 103 plants in 12∶12 photoperiods.

*ELF3* was first identified in Arabidopsis as an early flowering time mutant insensitive to photoperiod [31]. However, this gene has never been implicated as a regulator of the shade avoidance response. One way to investigate the effects of *ELF3* in this response is to perform a quantitative complementation test, analyzing the response in genetically similar lines carrying different dosages of *ELF3* alleles [38]. We performed a quantitative complementation test by crossing both HIF166L homozygous lines to the *elf3-1* mutant and Columbia wild type plants to generate F1 plants carrying Bay-0/*elf3-1*, Sha/*elf3-1*, Bay-0/Col, or Sha/Col alleles of *ELF3* (Figure 4). In terms of genotype, Col-0 *ELF3* shares the canonical allele at the non-synonymous polymorphism in the fourth exon with Bay-0 and has 7 fewer glutamines than Sha and 15 fewer than Bay-0. These hybrid plants were grown in simulated sun and shade both in 16∶8 and 12∶12 photoperiods, and measured for bolting and flowering time. In both photoperiods, plants carrying *ELF3*-Col-0 alleles presented a similar bolting and flowering response to shade regardless of the presence of *ELF3-Bay-0* or *ELF3-Sha*. On the contrary, significant differences were observed when *ELF3-Bay-0* and *ELF3-Sha* were the only functional *ELF3* alleles. In agreement with what we have shown before, plants carrying *ELF3-Bay-0* alleles present a stronger shade avoidance response than plants carrying *ELF3-Sha* alleles.

These experiments strongly suggest that Bay-0 and Sha alleles of *ELF3* induce different shade avoidance responses in Arabidopsis.

### HIF166L circadian rhythms

**Figure 5:**
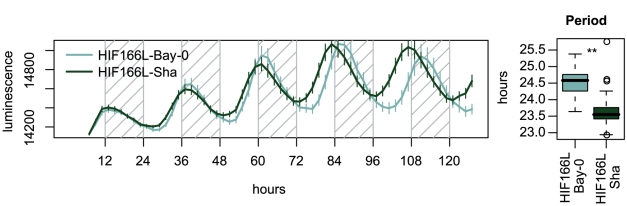
*SAR2* affects circadian period. Circadian rhythms in homozygous HIF166L plants. Homozygous progeny of the HIF166L line were transformed with a luciferase reporter gene driven by the CCR2 promoter. Luminescence rhythms of 44 HIF166L-Bay-0 and 77 HIF166L-Sha T1 plants were monitored under constant red light conditions using a CCD camera. Average luminescence rhythms ± standard errors are plotted against time for plants that presented a Relative Amplitude Error below 0.5. Shaded regions correspond to subjective nights. Box plot on the right represents period average estimates for the rhythms of the individual plants assayed. ** - ANOVA test p<0.001.

*ELF3* functions as an important regulator of flowering time and light input to the circadian clock [39]. Therefore, *ELF3* alleles in HIF166L-Bay and HIF166L-Sha could be altering circadian rhythms in addition to developmental time. To test this, we transformed the homozygous HIF166L lines with a luciferase reporter gene driven by the *COLD, CIRCADIAN RHYTHM, AND RNA BINDING 2* (*CCR2*; AT2G21660) promoter (CCR2::*luc*) [40]. Independent T1 plants were entrained in 12∶12 photoperiods for 6 days and released into constant red light, where circadian rhythms were measured by monitoring luminescence. Indeed, HIF166L lines carrying Bay-0 alleles had longer periods than the lines with the Sha allele (Figure 5). Therefore, different alleles of *ELF3* affect both developmental time phenotypes and circadian rhythms, although the relationship between these two phenotypes cannot be discerned from this experiment.

### *ELF3* complementation test

**Figure 6:**
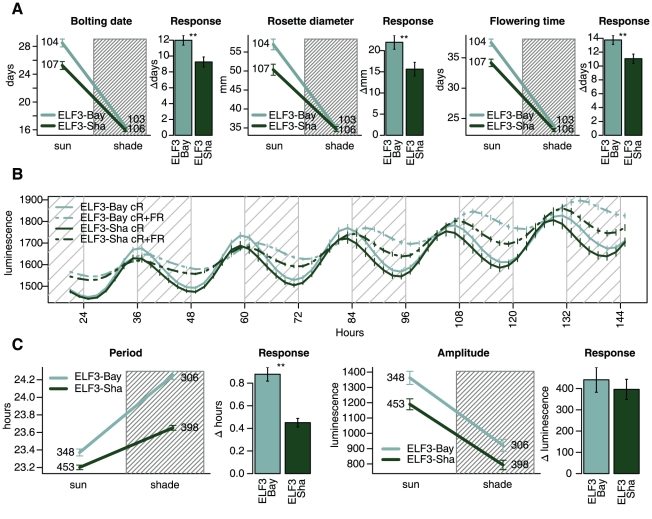
Rescued lines present differential response to shade in developmental time traits and circadian rhythms. Shade avoidance response phenotypes of *elf3-1* rescued lines. Mutant *elf3-1* plants with a stable insertion of CCR2::luc were transformed with the *ELF3-Bay* or *ELF3-Sha* genes driven by their own promoters. (A) T1 transformants were grown in 12∶12 under simulated sun or shade and measured for their shade avoidance response. Each data point represents the average ± standard error for each genotype and condition, and numbers beside each point represent the number of plants measured. Shaded versus non-shaded areas indicate measurements from plants in simulated shade and sun respectively. Bar plots show the increment ± standard error of each trait in simulated sun with respect to simulated shade. Asterisks on each bar plot indicate significant differences. ** - ANOVA test p<0.01. (B) and (C)- Luminescence rhythms of T1 transformants were monitored in 5 experiments under constant red light and 5 experiments under constant red plus far red light. (B) Circadian rhythms represent the average luminiscence ± standard error of 60–150 plants with a Relative Amplitude Error below 0.5. One representative experiment in each light condition is shown. Shaded regions correspond to subjective nights. (C) Line plots represent the average period or amplitude ± standard error for all 10 experiments performed. Numbers beside each line end represent the number of plants measured. Shaded versus non-shaded areas indicate measurements from plants in simulated shade and sun respectively. Bar plots represent the difference in period and amplitude between cR+FR and cR per genotype. ** - ANOVA test p<0.001.

Although *ELF3* is the strongest candidate for *SAR2*, one could argue that another of the 342 genes segregating in HIF166L could be interacting with the *elf3-1* allele to create the shade avoidance phenotypes observed in the quantitative complementation test. To rule out this possibility we cloned *ELF3-Bay* and *ELF3-Sha* alleles together with their own promoters and transformed them into *elf3-1* mutant background [31]. T1 plants carrying these constructs (*elf3-1* + *ELF3-Bay* and *elf3-1* + *ELF3-Sha*) were grown in simulated sun and shade environments and measured for their response to shade. Figure 6A shows that *elf3-1* + *ELF3-Bay* plants present significantly greater acceleration of bolting and flowering and greater reduction of rosette diameter than *elf3-1* + *ELF3-Sha* plants, as predicted by the QTL analysis. Unexpectedly, these transgenic plants showed more similar phenotypes in simulated shade than in simulated sun, opposite to what we had seen in HIF166L (Figure 2). Interactions between the *ELF3-Bay* and *ELF3-Sha* alleles with the Col genetic background of the *elf3-1* mutants could explain these differences.

If *ELF3* is responsible for the differences in the circadian rhythms in the homozygous HIF166L we should observe similar differences in the *elf3-1* + *ELF3-Bay* and *elf3-1* + *ELF3-Sha* transgenic lines. T1 plants from these lines containing the CCR2:luc reporter gene were entrained in white light 12∶12 cycles and monitored either under constant red light (cR) or in constant red plus far red light conditions (cR+FR) to simulate sun and shade environments. We observed an overall increase in the period and a decrease in the amplitude of the oscillations of the reporter gene in plants grown in the shade (Figures 6B and 6C). In concordance with the phenotypes observed in HIF166L, plants carrying *ELF3-Bay* alleles showed longer periods than plants carrying the *ELF3-Sha* allele. When comparing these period differences between alleles grown in both light conditions, we again see that plants with *ELF3-Bay* alleles have an increased response to low R:FR ratio. The ELF3-Bay alleles did not have a significant effect on the amplitude of the oscillations in response to shade (Figures 6B and 6C).

In summary, these transgenic experiments demonstrate that *ELF3* has an effect on the shade avoidance response and that Bay-0 and Sha alleles of this gene confer different responsiveness to the plants that carry them. Plants grown under simulated shade presented longer circadian periods and flowered earlier than plants grown under simulated sun, and the effect of Bay-0 alleles further increased circadian periodicity and shortened developmental time. Although it is temping to speculate that *ELF3* could determine the magnitude of the response to shade in the Bay-0 and Sha RIL population by modulating their circadian rhythms, we cannot discard the possibility of *ELF3* affecting both phenotypes independently.

## Discussion

In this work we detected *SAR2*, a QTL on chromosome 2 for the shade avoidance response in the Bay-0 and Sha RIL population of Arabidopsis. We developed a network analysis that integrated genomic information available for Arabidopsis, allowing us to propose a candidate gene for the *SAR2*. We have shown that different natural alleles of *ELF3*, the gene predicted by the network analysis, differentially modulate the shade avoidance response, confirming its identity as the gene responsible for *SAR2* in the Bay-0 and Sha RIL population.

### Shade avoidance response in the Bay-0 x Sha RIL population

We surveyed the shade avoidance response in adult plants grown in 12∶12 and 16∶8 photoperiods by measuring typical shade avoidance response traits [1]. Only leaf angle, a well-established shade avoidance trait [41], did not show significant changes across environments, probably due to the high variance of the measurements (data not shown). Leaf size traits, such as leaf blade width and length, were responsive to shade although they did not present significant variance among the Bay-0 x Sha RILs (Figure S1). QTL analysis on the remaining traits resulted in distinct QTL profiles for plant size traits and developmental time traits, suggesting different underlying genetic mechanisms for each group of phenotypes (Figures 1 and S3). Although rosette diameter may seem a measurement of plant size rather than developmental time the overlapping QTLs between this trait and other developmental time traits should not be surprising, since rosette diameter was measured at bolting time, and plants that take more time to bolt will grow bigger (Figures 1 and S3).

The majority of QTLs were in common between the sun and the foliar shade environments, suggesting that most variation in these traits is controlled by loci that act independently of these light qualities. Previous QTL analyses on the same RIL population in high versus low density planting in natural environments resulted in the detection of a greater number of QTLs, which included *SAR2* and our developmental time QTLs in chromosome 1, 4 and 5 (Figure S3 and [14]). It is quite possible that the effect of the natural environments and crowding in those experiments, versus our homogeneous light and temperature conditions, are responsible for the differences between these analyses.

Comparison of the results from the QTL analysis performed in 16∶8 and 12∶12 photoperiods showed colocalization of the major QTLs in both photoperiods, although the size of the effects was always smaller in 16∶8 photoperiods, possibly due to the smaller number of lines assayed in 16∶8 (126) in comparison to the 12∶12 environment (253). On the other hand, the number and positions of minor QTLs differ, suggesting a number of photoperiod-specific loci affecting developmental time (see QTLs in simulated sun and shade on chromosomes 3 and 5, Figures S3 and S5). Previous analysis in the same RIL population performed in 16∶8 and 8∶16 photoperiods found similar results [14], [42].

### Network analysis

One of the benefits of working with Arabidopsis, and especially with the Bay-0 and Sha RIL population, is the wealth of genomic resources available [20], [22], [43], [44]. The network analysis proposed here makes use of genome-wide datasets to identify genes potentially regulating other genes in their pathways. This analysis resulted in the identification of *ELF3* as a likely regulator of genes involved in circadian rhythms and/or flowering time such as *EARLY FLOWERING 4* (*ELF4*; AT2G40080), *CCR2* and *SNF1-RELATED PROTEIN KINASE 2-2* (*SnRK2.*2; AT3G50500) (Figure 3) [45]–[47].

Our network method is largely based on genome-wide expression profiles (steps 1 and 3), and therefore it mainly identifies genes that exert their effect through alteration of the expression patterns in their pathways. Although one could imagine that a good number of the polymorphisms that have phenotypic consequences could directly or indirectly alter the expression of downstream genes, by no means is this always the case. The shade avoidance response is a complex process that has pleiotropic effects in the plant, therefore expression network modulation was expected. In cases where more specific phenotypes are under study, other kinds of data, such as protein-protein interaction, metabolic pathways or literature mining, can be added or used to construct the network. To illustrate this point, we applied the network analysis to *5*′*ADENYLYLPHOSPHOSULFATE REDUCTASE 2* (*APR2*; AT1G62180), a gene encoding a phosphosulfate reductase for which Sha has a reduced function allele that leads to high sulfate content [48]. Network examination on the location of this gene did not detect *APR2* as a regulatory hub, probably due to its enzymatic function (data not shown).

A major concern during the construction of our network method was the inability to identify uncharacterized genes. Since we used the functional classification of each candidate gene to identify significant connections with its partners, an uncharacterized gene will show no links. A good example illustrating this flaw is that our network analysis was not able to identify AT5G43630, a gene underlying a QTL for sulfate content in the Bay-0 and Sha RIL population [49]. Due to its recent annotation, AT5G43630 was not included in the ATH1 microarrays and was therefore absent from the co-expression databases used in this work. As genome wide information becomes more abundant and annotation improves, this issue will be of lesser concern. Again, any kind of relational evidence can be used during this step.

Although this network analysis will certainly not identify every causative polymorphism with an effect on a QTL, this methodology could be easily adapted to include any kind of relevant genome-wide datasets and may prove very useful in many QTL studies.

### *ELF3* and the shade avoidance response

We have found here for the first time that ELF3 is involved in the regulation of flowering time and circadian rhythms in response to shade (Figure 6). ELF3′s relation to shade could be through its interaction with the red light photoreceptor phyB [39], the main photoreceptor involved in the shade avoidance response [3]. However, other possibilities need to be considered since ELF3 has been shown to regulate flowering time independently of phyB and to function downstream of the blue light photoreceptor CRY2 [39], [50], [51], which controls cotyledon unfolding in response to far-red light [17]. Additional photoreceptors, such as phyD and phyE, have been implicated in the shade avoidance response and could also modulate the effect of ELF3 [4].

Our experiments show that natural alleles of *ELF3* differentially regulate circadian period and flowering time in response to shade (Figure 6). ELF3 is a highly conserved plant specific nuclear protein that has been suggested to be part of the central clock oscillator and to act as a link between light and the circadian clock [39], [52]–[55]. In addition, ELF3 affects flowering time in a photoperiod dependent manner through the classical *GIGANTEA* (*GI*; AT1G22770) and *CONSTANS* (CO, AT5G15840) pathway, but also through alternative pathways such as *SHORT VEGETATIVE PHASE* (SVP; AT2G22540) and *FLOWERING LOCUS C* (FLC; AT5G10140) [31], [51], [56].

Recent findings show that the shade avoidance response in flowering time is dependent of long day photoperiods and of functional *GI* and *CO* [4], [57]. Wollenberg *et al*. showed that the shade treatment delays the peak expression of GI, which is in agreement with the delay in CCR2::luc rhythms that we observe under low R:FR conditions. Interestingly, we found that *ELF3-Bay-0*, which promoted stronger shade avoidance response, also promoted a larger delay in *CCR2::luc* rhythms (Figure 6). It is thus possible that to accelerate flowering in response to shade, ELF3 delays the rhythmicity of GI through modulation of the circadian clock. However, it has been shown that ELF3 affects GI protein stability through direct binding, indicating that changes in circadian rhythms and flowering time could be independent from one another [51]. In fact, the promiscuous nature of ELF3 and the pleiotropic character of the shade avoidance response suggest a complex regulation of pathways in both cases.

We have found that *ELF3* harbors natural polymorphisms determining its response to the shade treatment, which as an adaptive trait can affect the fitness of natural populations in their environments [12]. In our work the Bay-0 allele of *ELF3* conferred a greater response to the shade treatment than the Sha allele. The Arabidopsis accession Bay-0 was collected from a set-aside crop field in Germany while the Sha natural population grows at high altitude in the Pamir mountains (Tajikistan) [42]. Stronger shade avoidance responses are expected and have been reported in plants from competitive environments such as arable land in comparison with alpine plants that grown surrounded by sparse vegetation [58]. Although our findings support this hypothesis, additional experiments are needed to confirm the effect of *ELF3* under these particular environments.

In summary, we have shown here that a network analysis approach utilizing multiple genomic databases can be a highly effective tool to identify causative genes in QTL analyses. Using this network approach we have identified *ELF3* as the candidate gene for a QTL affecting the shade avoidance response in the Bay-0 and Sha RIL population. We have proved that the Bay-0 and Sha alleles of *ELF3* differentially affect the shade avoidance response in circadian rhythms and developmental time traits.

## Materials and Methods

### Plant material

The Bay and Sha core RIL population as well as HIF144 and HIF166 were kindly provided by Dr. Olivier Loudet (INRA, Versailles, France). HIF144 and HIF166 are lines derived from F6 Bay-0 and Sha RILs that maintain residual heterozygosity in localized regions of their genomes. Luciferase expressing HIF166L-Bay and HIF166L-Sha T1 lines were obtained by *Agrobacterium tumefaciens* transformation of the homozygous HIF166L lines with CCR2::luc [40].

The *elf3-1* mutant line has an EMS mutation in the Columbia background that causes an early stop codon and behaves like null alleles [31]. These plants also carry an additional mutation in *gl1* [50]. A stable *elf3-1* line with CCR2::luc was obtained from Dr. Stacey Harmer and Dr. Michael Covington. This line was transformed using *Agrobacterium tumefaciens* containing the Bay-0 and Sha alleles of *ELF3* together with 1.5 kb of their corresponding promoter region into the pJIHOON212 vector.

We reciprocally crossed Col-0 and *elf3-1* plants to the HIF166L-Bay and HIF166L-Sha homozygous lines to obtain F1 lines for the quantitative complementation test. No statistically significant phenotypic differences were observed between reciprocal crosses.

### Plant growth

All plants were grown in 16∶8 photoperiods unless stated. All developmental time experiments were performed by stratifying seeds for 4 days followed by planting in flats of 35 60 mm^2^ square pots using randomized block designs. After 5 days of incubation under white light, flats were positioned in Conviron chambers equipped with white and far-red fluorescent lights to simulate sun and shade environments. Red (655–665 nm) to far-red (725–735 nm) ratios in simulated sun ranged between 2.3 and 2.8 and between 0.50 and 0.58 in simulated shade. Photosynthetically active radiation ranged between 90 µmol m^−2^ s^−1^ and 97.1 µmol m^−2^ s^−1^, with an average of 94.8 µmol m^−2^ s. Temperature was kept constant at 20°C. The positions of the flats were changed in the growth chamber every 2 to 3 days to reduce light or temperature biases. To facilitate plant identification, each plant was assigned a barcoded tag indicating the genotype, position in the growth chamber, position in the flat and treatment.

For the circadian rhythm experiments, T1 seedlings of the appropriate genotypes were plated on MS medium without sucrose and with the appropriate antibiotic, stratified for 4 days (4°C, dark), and entrained in 12∶12 photoperiods for 7 days. After entrainment, plants were transferred to new MS plates with antibiotics and moved to red light or red + far-red light conditions, where luminescence was recorded.

Similar stratification and entrainment protocol as for the circadian rhythms experiments was followed for the transgenic experiment with rescued *elf3-1* plants, but after entrainment for 7 days resistant lines were transplanted to soil.

### Developmental time measurement

Petiole length, blade length and blade width were measured 38 days after germination using an electronic caliper. For leaf blade length and width the longest possible measurement was made in leaves 4 and 6. Leaf angle was measured 38 days after germination using an electronic protractor as the angle formed by the petiole of the leaf and the soil. Rosette diameter, bolting date and leaf number were measured when the rising meristem separated from the rosette. Rosette diameter was measured as the widest diameter found in the plant. This trait was not measured in the F1 plants used for the quantitative complementation test due to their increased size caused by hybrid vigor [59]. Flowering time was recorded as the day of the first open flower.

### Statistical analyses

We obtained trait indexes for each RIL in the sun and shade environments fitting mixed effect models using the lme4 package in R [60], [61]. These mixed effect models included treatment as a fixed effect and genotype, genotype x treatment, chamber, shelf, flat, row and column in the flat, and person measuring as random effects when significant. For each RIL, two shade avoidance response indices were calculated. For the subtraction index the indices in the shade for each RIL were subtracted to the indices in the sun. The residual indices correspond to the residuals from a regression of the trait indices in the shade on the sun indices. The signs of the residuals from this regression were reversed to obtain a more intuitive shade avoidance index in which higher values represent stronger shade avoidance responses. Phenotypic measurements whose distributions did not fit a normal distribution according to the Shapiro-Wilks test (p<0.01) were transformed by taking the natural logarithm of each phenotype. All indices were subsequently calculated as for the untransformed traits.

### QTL analysis

We performed QTL analysis of the sun and shade indexes as well as of the shade avoidance response indexes for each phenotypic trait in each of the 253 RILs. The genetic map for the Bay-0 x Sha RIL population consisted of 578 SFPs and microsatellites as described before [35], [42]. QTL analysis was performed with the R/qtl package in R with the Interval Mapping (IM) and Composite Interval Mapping methods, obtaining similar results [61]–[65]. LOD thresholds for significance of QTLs were estimated using 10,000 permutations of the phenotypic data. QTL analyses for the log-transformed datasets were similar to those for the non transformed datasets. QTL analyses of the subtraction and residual shade avoidance response indices also presented similar results.

To select a subset of lines for repeating the QTL experiment in long days, we created a custom script in R that performed QTL analysis on 126 RILs randomly sampled from our population of 253 lines. After 25,000 permutations, the set of 126 lines that maximized the effect in *SAR2* was used in the LD experiment.

### Network analysis

Network analysis was performed separately using the 363 candidate genes included in the interval starting from gene AT2G24100 to AT2G27500 and in a subset of 133 candidate genes between AT2G25360 and AT2G26640 according to TAIR version 9 [43]. Co-expression data for each gene was obtained from ATTED-II version c4.1 that was calculated including 1388 microarray experiments [20]. For each candidate gene we created an undirected network that linked each candidate gene to genes with correlation mutual ranks below 50 and over the maximum rank for that gene minus 50 [20]. Next, we narrowed the network and established directionality by removing co-expressed genes that did not have an eQTL in the position of the candidate gene according to the Bay-0 x Sha eQTL analysis [22]. Thus, we considered only those co-expressed genes whose expression levels are segregating in the Bay-0 and Sha RIL population and are differentially controlled by alleles located in the region of the candidate genes. eQTL confidence intervals were calculated as the interval where the eQTL has its maximum together with its contiguous intervals [22]. For each candidate gene in our network we looked for genes involved in similar processes by counting the number of co-expressed genes that shared one or more GO Slim terms with the candidate gene [19]. GO categories describe aspects of a gene product's biology by assigning genes to cellular compartments, molecular functions and biological processes, and GO Slim terms are a subset of GO terms which give a broad overview of the ontology content without the detail of the specific fine grained GO terms [19]. GO Slim terms were obtained from the GO SLIM dataset from TAIR, version of the 22nd of January 2010. Only GO terms under the relationship ‘involved in’, which imply biological processes, were used. Finally, we marked as polymorphic those genes that presented non-synonymous polymorphisms between Bay-0 and Sha according to [44] or had a cis-eQTL according to [22]. Custom R and Perl scripts carried out all these processes, and the results were plotted using Cytoscape [66].

### HIF and natural population genotyping

Leaves from each plant in the fine mapping experiments were collected after the date of flowering and frozen at −80°C. DNA was extracted using the Promega DNA Purification System. Touchdown PCR was performed on this DNA in a MJ Research PTC-200 Thermocycler with a starting annealing temperature of 58°C, which decreased 0.5°C per cycle for 15 cycles and stayed constant at 55°C for 30 cycles. Extension time was 40 seconds and denaturizing steps were performed for 30 seconds at 96°C. PCR products were run in 3% agarose gel stained with EtBr and the genotype of the plant was assessed. The primers used for the PCRs were: MSAT2.36, F - CCAAGAACTCAAAACCGTT, R – GATCTGCCTCTTGATCAGC; oJM88/89, F – TCTTCACTTCCCCCAAGCGTTAC, R – CCTTGAGGCAATGAACATCGGC; oJM29/30, F – ATCAAGCAGAAGAAGAAACAAGAA, R – GCAGGTGAAAACTGAATAGAACTT; oJM34/35, F – GCAAATGAATGGACTTGATGGTT,R – ACAGGGATTGGGCGGTGATGG; oJM40/77, F – CCTCCTGGTAATGGCTACTTCCC, R – ATTCTGGCAGCATTCTCACTCG; oJM41/42, F- GCTAACTCTGTGATGGCAACCG, R – ATTAGGGCGTGAAAGCGACTG; nga1126, F – CGCTACGCTTTTCGGTAAAG, R – GCACAGTCCAAGTCACAACC; oJM94/95, F – TCTTCTTCGTCTCTTTGGGCTTCG, R – GATTTTAAGAAGAAGAATGCGGGG; oJM96/97, F – CACACATAACAACAGACCCACTTCG, R – CGAAGGAGGGTTTGGTTGCG; MSAT2.7, F – CTCAAATCAAGAACGCTGAC, R – CCCGATATAGACAACGACAA. All markers show insertion or deletion polymorphisms between Bay-0 and Sha and their positions are indicated in Figure 2. Genotyping of Arabidopsis natural populations was performed using primer pair oJM40/77, which targets the tri-nucleotide polymorphism in ELF3.

### Circadian rhythm estimation

All plants assayed for rhythmicity carried the luciferase gene driven by the circadian regulated promoter of the CCR2 gene (CCR2::luc)[40]. In all cases plants were entrained as described above and imaged under constant conditions for 6 to 7 days. Constant red light (R, total PAR 64 uE) or constant red plus far red light (R+FR, total PAR 64uE with a R:FR ratio of 0.5) conditions were created with LED lights. Plants were monitored using a CCD camera taking pictures every 2 hours. The data collected was analyzed for rhythmicity using the luciferase activity method described in [67]. Only rhythms with a Relative Amplitude Error below 0.5 were considered for the analysis. Circadian rhythm plots show average luminescence rhythms ± standard errors against time. Shaded regions in these graphs correspond to subjective nights.

## Supporting Information

Figure S1 Phenotypic distribution for unresponsive traits. Phenotypic distributions of traits that did not show significant treatment or line by treatment shade avoidance response among the Bay-0 x Sha RILs grown in 12∶12 photoperiods. Lines plot the density of the distribution of the sun, shade and shade avoidance response residual indices calculated as detailed in Materials and Methods. Closed circles and squares represent the estimated values for the Bay-0 and Sha parental strains respectively.
(0.36 MB PDF)

Figure S2 Phenotypic distribution for responsive traits. Phenotypic distributions of traits that showed significant line by treatment effects among the Bay-0 x Sha RILs. Line plot the density of the distribution of the sun, shade and shade avoidance response residual indices calculated as detailed in Materials and Methods. Leaf number and petiole length were measured only in 12∶12 photoperiods.
(0.46 MB PDF)

Figure S3 QTL analysis in simulated sun and simulated shade in 12_12 photoperiods. Results from the QTL analysis in simulated sun and shade under 12∶12 photoperiods. X-axis represents each of the 5 chromosomes of Arabidopsis, tick marks in the axis represent markers used in the genetic map. LOD score is represented in the y-axis. Representative estimations of the LOD thresholds are illustrated by horizontal dotted lines (Simulated sun, average  = 2.72, range  = 2.62-2.82; Simulated shade, average  = 2.67, range  = 2.53–2.80).
(0.78 MB PDF)

Figure S4 Additive effect of *SAR2*. Additive effects (a, y-axis) are estimated as half the difference between the phenotypic averages of the residuals indices for the Bay-0 and Sha homozygotes. Positive numbers indicate that Bay-0 alleles increase the shade avoidance response with respect to Sha. Only chromosome 2 is represented. Additive effects ± standard errors are represented as a line enclosed in a light colored region. Blue and green lines represent additive effects in 12∶12 and 16∶8 respectively.
(0.30 MB PDF)

Figure S5 QTL analysis of the shade avoidance response in 16_8 photoperiods. QTL results for the Bay-0 and Sha RILs grown in long day photoperiods (16∶8) in simulated sun, simulated shade and for the shade avoidance response residual index. X-axis represents each of the 5 chromosomes of Arabidopsis, tick marks in the x-axis represent markers used from the genetic map. LOD score is represented in the y-axis. A representative estimation of the LOD threshold is illustrated by a horizontal dotted line.
(0.70 MB PDF)

Figure S6 HIF144 and HIF166 phenotypes under simulated shade. Phenotypes of HIF lines segregating for *SAR2*. A) HIF lines 144 and 166 are heterozygous for all or part of the confidence interval of the QTL in chromosome 2. Each horizontal line represents the region of interest in chromosome 2. Circles represent molecular markers used to genotype the plants. Numbers on top of the chromosomes indicate positions in megabases in the AGI map (TAIR 9). The colored boxed area represents the 2-LOD confidence interval for *SAR2*. B) Barplots represent bolting, flowering and rosette diameter average phenotypes measured in the progeny of the HIFs depicted in (A) grown under simulated shade. Names and numbers under each bar indicate genotype and number of plants assayed. Different letters on top of each bar represent significant differences between genotypes (p<0.05, Tukey's HSD test).
(0.31 MB PDF)

Figure S7 Network analysis. Network analysis for the 363 genes located in the union of *SAR2*'*s* confidence interval and HIF166-L heterozygous interval. Nodes represent genes. Only nodes with at least one edge are represented. Nodes with thick border are the candidate genes located in the interval. Edges connect genes that are co-expressed with the candidate gene and have an eQTL in the position of the candidate gene. Edges returning to the candidate genes represent cis eQTLs. Edges colored in red connect genes that share one or more functional category. Red color nodes are genes with polymorphisms between Bay-0 and Sha. The node representing the *ELF3* gene, which has more connections to functionally related genes than any other node in the network, is enclosed in a colored box.
(3.21 MB PDF)

Figure S8 *ELF3* polymorphisms. *ELF3* polymorphic regions between Bay-0 and Sha. A) Polymorphisms found in the coding region and 1.5 kb upstream of the Bay-0 and Sha alleles of *ELF3* are indicated. Non-synonymous polymorphisms are indicated with the amino-acid changes found in parenthesis. B) Poly-Q insert size variation among natural populations of Arabidopsis. Marker bands have a size of 500 bp. 1- Kz-9, 2- est-1, 3- Bor-1, 4- NFA-10, 5- Bor 4, 6- NFA-8, 7- c24, 8- Nd-1, 9- sq-8, 10- Ler1, 11- wa-1, 12- Mz 0, 13- fer-0, 14- Van 0, 15- lp2-2, 16- Gu-0, 17- Lz-0, 18- Mr0, 19- Zdr-1, 20- ct-1, 21- Hr10, 22- Ra-0, 23- Uod-1, 24- Ws-0, 25- Col-0, 26- Ren-11, 27- An-1, 28- Wei-0, 29- Oy-0, 30- Se-0, 31- Wt-5, 32- Zdr-6, 33- Lp2-6, 34- Pu 2-7, 35- HR-5, 36- Gy-0, 37- Sorbo, 38- Nok-3, 39- Ull-2-3, 40- Pna-10, 41- Var 2-6, 42- Knox-18, 43- RRS-7, 44- RRS-10, 45- Kas 1, 46- Br-0, 47- CIBC5, 48- Kondara, 49- Ag-0, 50- Kas-1, 51- Ms-0, 52- Omo2-3, 53- CS22491, 54- Bur-0, 55- Knox-10, 56- Bor-1, 57- Kas-1, 58- CIBC-17, 59- Bill 7, 60- Wt-5, 61- Ts-5, 62- HR-5, 63- Pna 17, 64- Uod-7, 65- Var 2-1, 66- Sha, 67- Ts-1, 68- Ws-2, 69- Kin-0, 70- Rmx-A180, 71- Tsu-1, 72- Fab-4, 73- Mrk-0, 74- Lov-5, 75- Ren-1, 77- Pu 2-2-3.
(0.47 MB PDF)
