# Supplementary figures and images for: Network Analysis Identifies ELF3 as a QTL for the Shade Avoidance Response in Arabidopsis
Source: PLoS Genet. 2010 Sep 9;6(9):e1001100. doi: 10.1371/journal.pgen.1001100 (PMC2936530; doi:10.1371/journal.pgen.1001100)

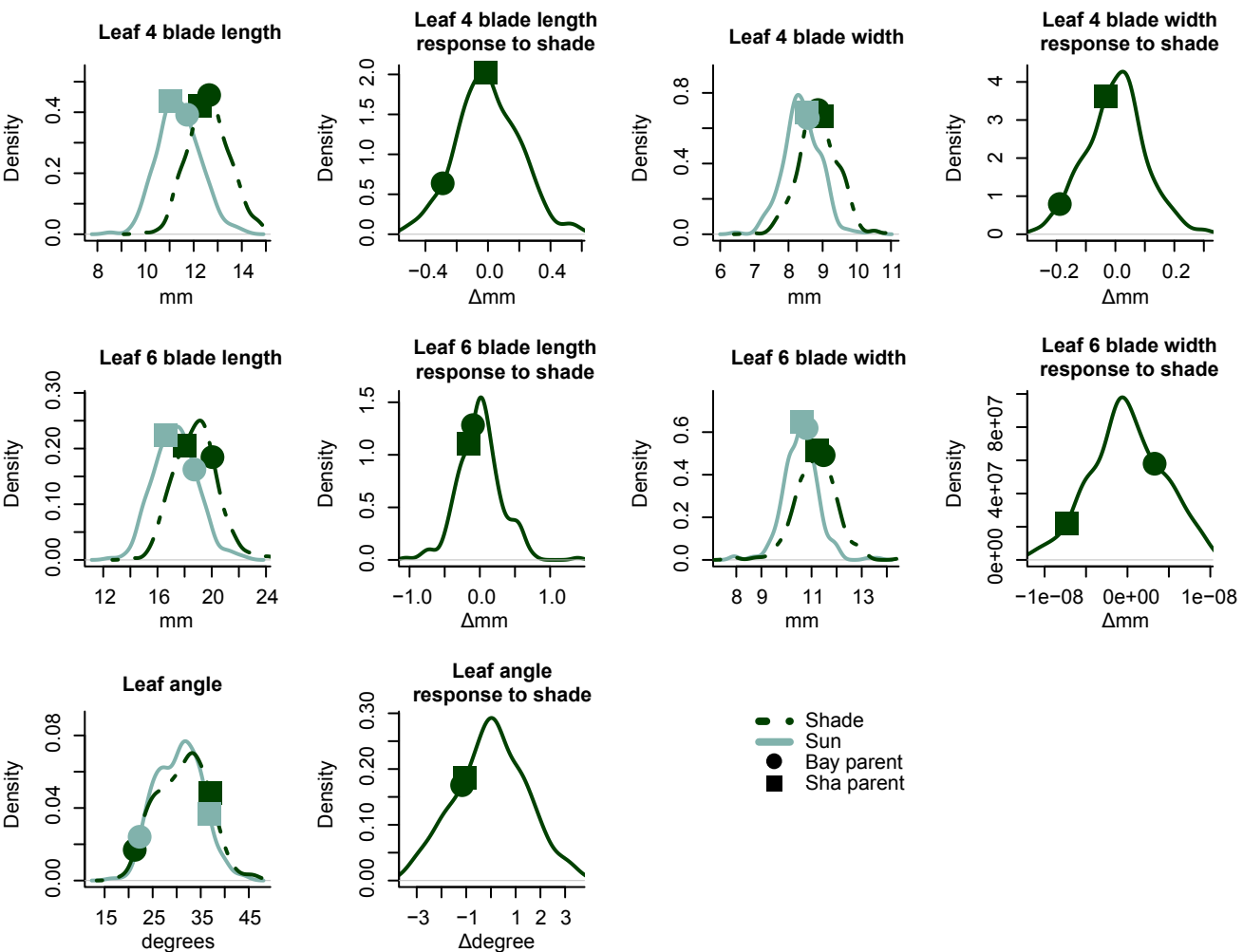

Supplement: Figure S1 — Phenotypic distribution for unresponsive traits. Phenotypic distributions of traits that did not show significant treatment or line by treatment shade avoidance response among the Bay-0 x Sha RILs grown in 12∶12 photoperiods. Lines plot the density of the distribution of the sun, shade and shade avoidance response residual indices calculated as detailed in Materials and Methods. Closed circles and squares represent the estimated values for the Bay-0 and Sha parental strains respectively. (0.36 MB PDF) [file pgen.1001100.s001.pdf]

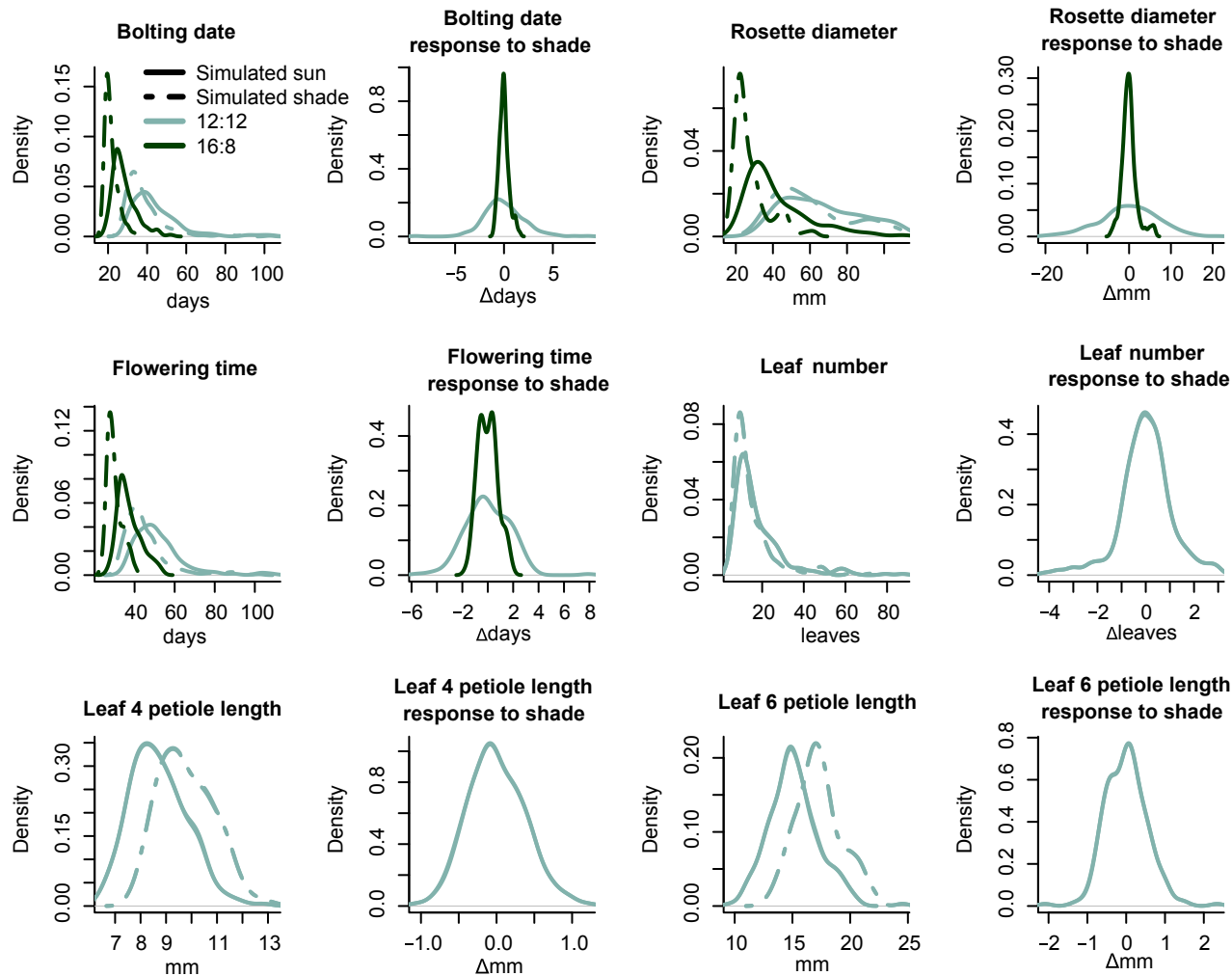

Supplement: Figure S2 — Phenotypic distribution for responsive traits. Phenotypic distributions of traits that showed significant line by treatment effects among the Bay-0 x Sha RILs. Line plot the density of the distribution of the sun, shade and shade avoidance response residual indices calculated as detailed in Materials and Methods. Leaf number and petiole length were measured only in 12∶12 photoperiods. (0.46 MB PDF) [file pgen.1001100.s002.pdf]

**Simulated sun**

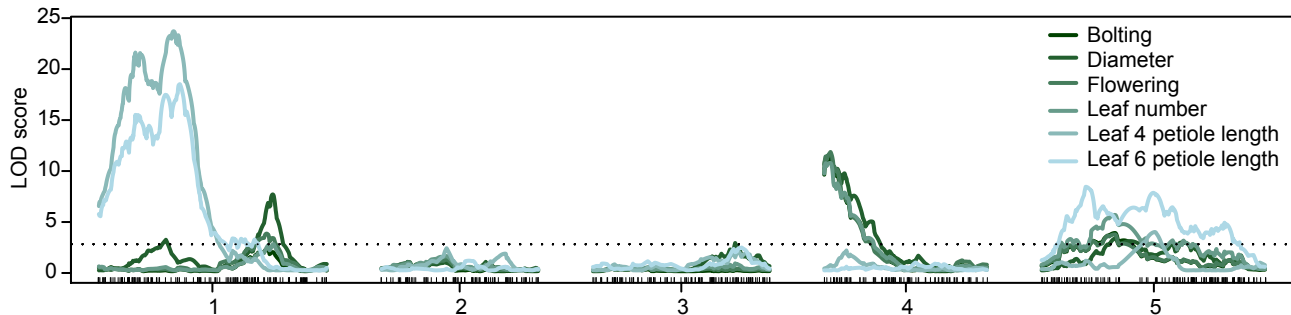

**Simulated shade**

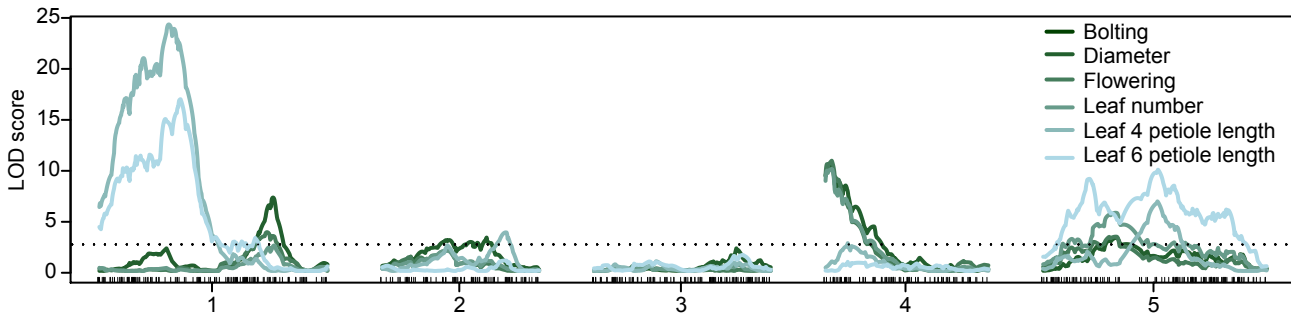

Supplement: Figure S3 — QTL analysis in simulated sun and simulated shade in 12_12 photoperiods. Results from the QTL analysis in simulated sun and shade under 12∶12 photoperiods. X-axis represents each of the 5 chromosomes of Arabidopsis, tick marks in the axis represent markers used in the genetic map. LOD score is represented in the y-axis. Representative estimations of the LOD thresholds are illustrated by horizontal dotted lines (Simulated sun, average = 2.72, range = 2.62-2.82; Simulated shade, average = 2.67, range = 2.53–2.80). (0.78 MB PDF) [file pgen.1001100.s003.pdf]

simulated sun

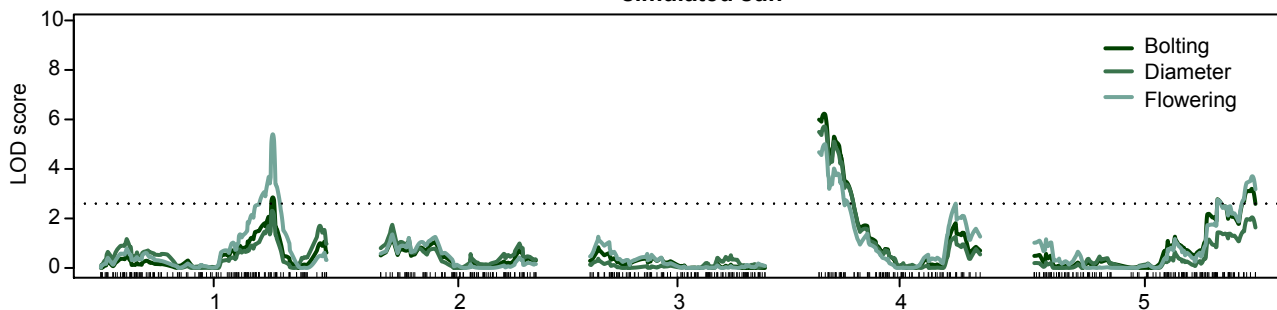

simulated shade

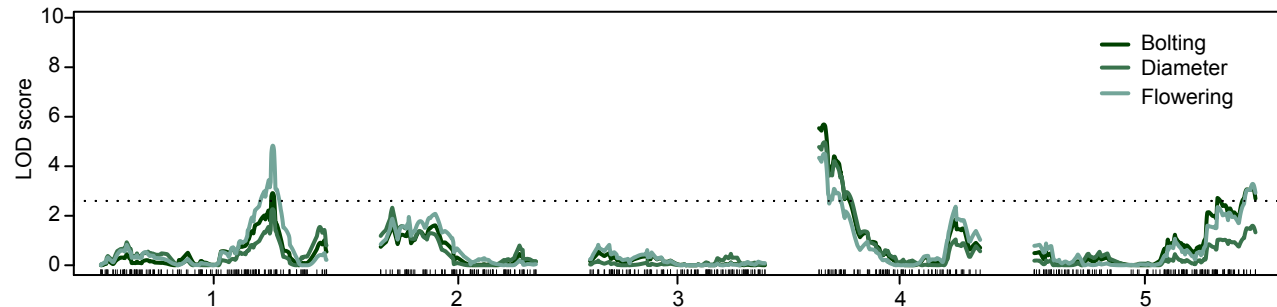

response to shade

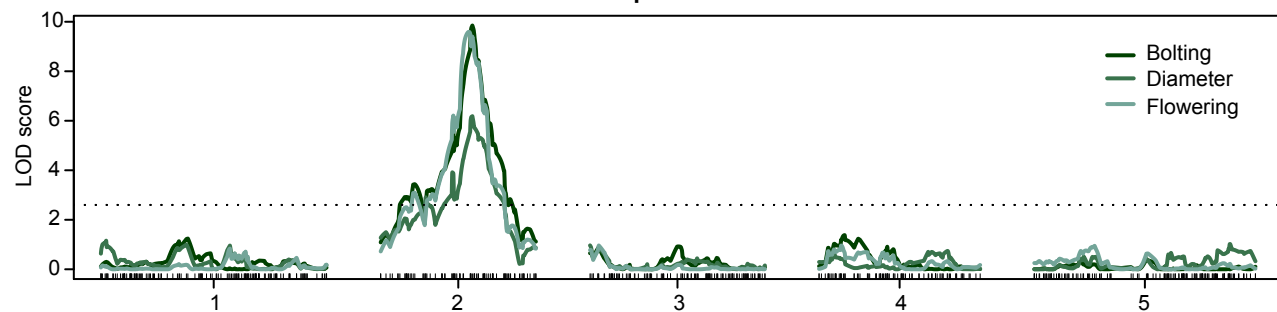

Supplement: Figure S5 — QTL analysis of the shade avoidance response in 16_8 photoperiods. QTL results for the Bay-0 and Sha RILs grown in long day photoperiods (16∶8) in simulated sun, simulated shade and for the shade avoidance response residual index. X-axis represents each of the 5 chromosomes of Arabidopsis, tick marks in the x-axis represent markers used from the genetic map. LOD score is represented in the y-axis. A representative estimation of the LOD threshold is illustrated by a horizontal dotted line. (0.70 MB PDF) [file pgen.1001100.s005.pdf]

**A**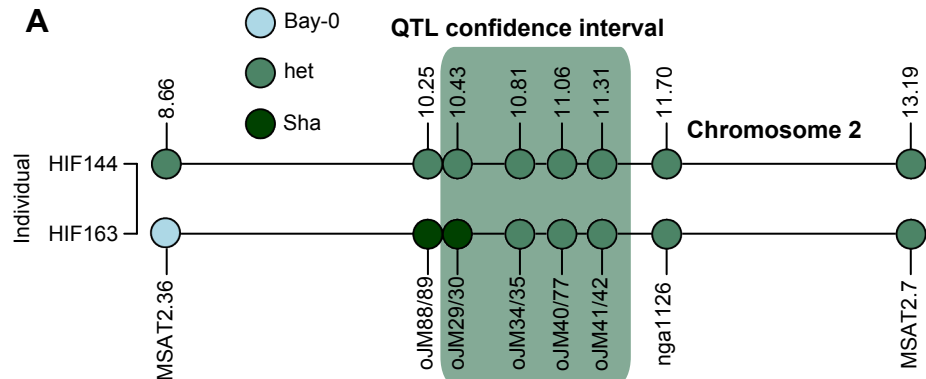**B**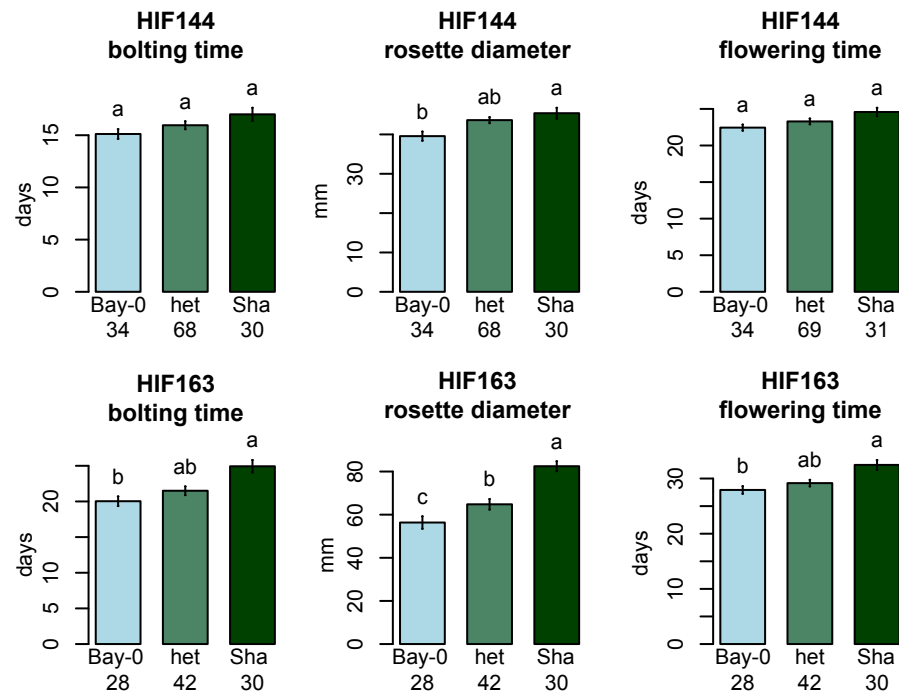

Supplement: Figure S6 — HIF144 and HIF166 phenotypes under simulated shade. Phenotypes of HIF lines segregating for SAR2. A) HIF lines 144 and 166 are heterozygous for all or part of the confidence interval of the QTL in chromosome 2. Each horizontal line represents the region of interest in chromosome 2. Circles represent molecular markers used to genotype the plants. Numbers on top of the chromosomes indicate positions in megabases in the AGI map (TAIR 9). The colored boxed area represents the 2-LOD confidence interval for SAR2. B) Barplots represent bolting, flowering and rosette diameter average phenotypes measured in the progeny of the HIFs depicted in (A) grown under simulated shade. Names and numbers under each bar indicate genotype and number of plants assayed. Different letters on top of each bar represent significant differences between genotypes (p<0.05, Tukey's HSD test). (0.31 MB PDF) [file pgen.1001100.s006.pdf]

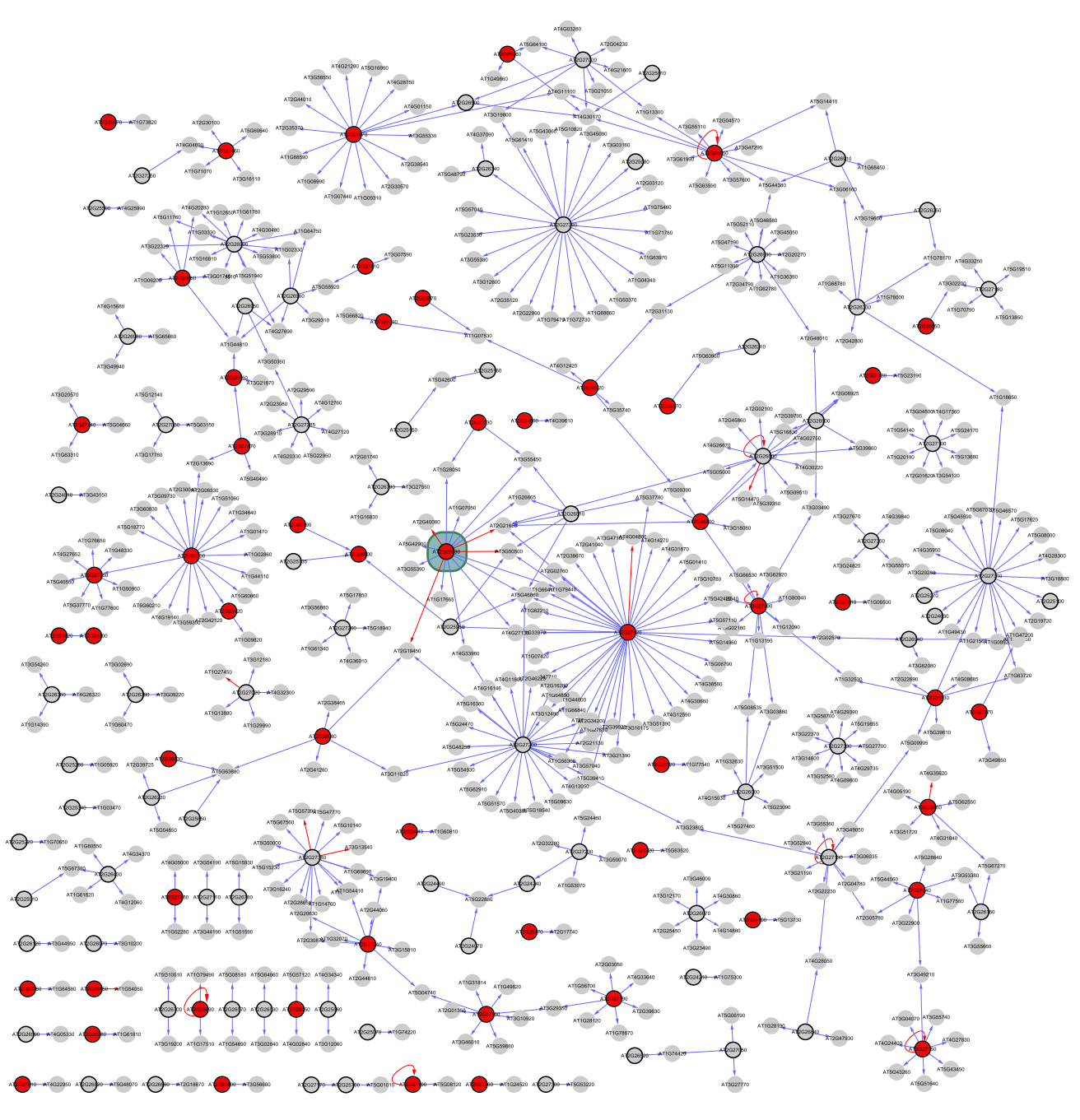

Supplement: Figure S7 — Network analysis. Network analysis for the 363 genes located in the union of SAR2's confidence interval and HIF166-L heterozygous interval. Nodes represent genes. Only nodes with at least one edge are represented. Nodes with thick border are the candidate genes located in the interval. Edges connect genes that are co-expressed with the candidate gene and have an eQTL in the position of the candidate gene. Edges returning to the candidate genes represent cis eQTLs. Edges colored in red connect genes that share one or more functional category. Red color nodes are genes with polymorphisms between Bay-0 and Sha. The node representing the ELF3 gene, which has more connections to functionally related genes than any other node in the network, is enclosed in a colored box. (3.21 MB PDF) [file pgen.1001100.s007.pdf]

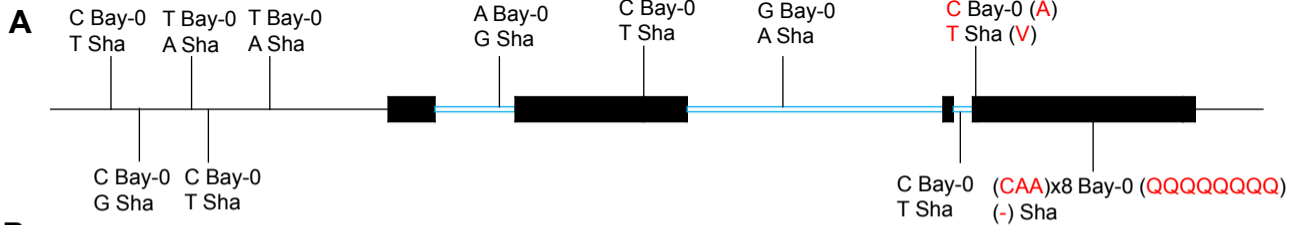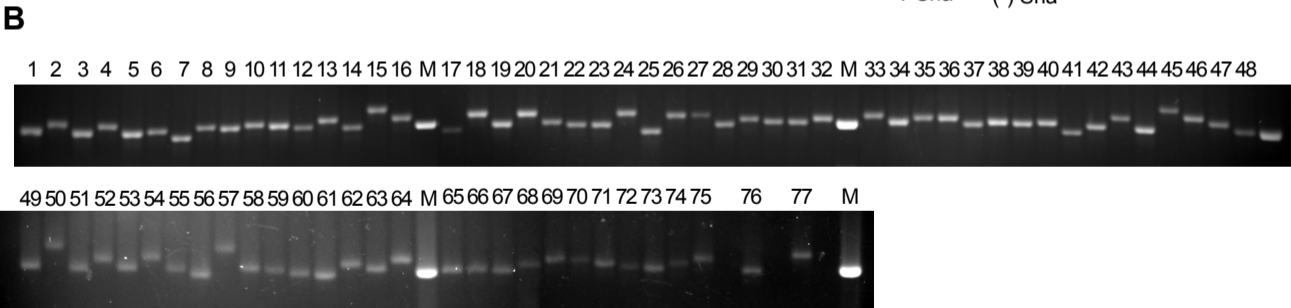

Supplement: Figure S8 — ELF3 polymorphisms. ELF3 polymorphic regions between Bay-0 and Sha. A) Polymorphisms found in the coding region and 1.5 kb upstream of the Bay-0 and Sha alleles of ELF3 are indicated. Non-synonymous polymorphisms are indicated with the amino-acid changes found in parenthesis. B) Poly-Q insert size variation among natural populations of Arabidopsis. Marker bands have a size of 500 bp. 1- Kz-9, 2- est-1, 3- Bor-1, 4- NFA-10, 5- Bor 4, 6- NFA-8, 7- c24, 8- Nd-1, 9- sq-8, 10- Ler1, 11- wa-1, 12- Mz 0, 13- fer-0, 14- Van 0, 15- lp2-2, 16- Gu-0, 17- Lz-0, 18- Mr0, 19- Zdr-1, 20- ct-1, 21- Hr10, 22- Ra-0, 23- Uod-1, 24- Ws-0, 25- Col-0, 26- Ren-11, 27- An-1, 28- Wei-0, 29- Oy-0, 30- Se-0, 31- Wt-5, 32- Zdr-6, 33- Lp2-6, 34- Pu 2-7, 35- HR-5, 36- Gy-0, 37- Sorbo, 38- Nok-3, 39- Ull-2-3, 40- Pna-10, 41- Var 2-6, 42- Knox-18, 43- RRS-7, 44- RRS-10, 45- Kas 1, 46- Br-0, 47- CIBC5, 48- Kondara, 49- Ag-0, 50- Kas-1, 51- Ms-0, 52- Omo2-3, 53- CS22491, 54- Bur-0, 55- Knox-10, 56- Bor-1, 57- Kas-1, 58- CIBC-17, 59- Bill 7, 60- Wt-5, 61- Ts-5, 62- HR-5, 63- Pna 17, 64- Uod-7, 65- Var 2-1, 66- Sha, 67- Ts-1, 68- Ws-2, 69- Kin-0, 70- Rmx-A180, 71- Tsu-1, 72- Fab-4, 73- Mrk-0, 74- Lov-5, 75- Ren-1, 77- Pu 2-2-3. (0.47 MB PDF) [file pgen.1001100.s008.pdf]
