# Supplementary material for: Network Analysis Identifies ELF3 as a QTL for the Shade Avoidance Response in Arabidopsis
Source: PLoS Genet. 2010 Sep 9;6(9):e1001100. doi: 10.1371/journal.pgen.1001100 (PMC2936530; doi:10.1371/journal.pgen.1001100)
Supplement: Figure S4 — Additive effect of SAR2. Additive effects (a, y-axis) are estimated as half the difference between the phenotypic averages of the residuals indices for the Bay-0 and Sha homozygotes. Positive numbers indicate that Bay-0 alleles increase the shade avoidance response with respect to Sha. Only chromosome 2 is represented. Additive effects ± standard errors are represented as a line enclosed in a light colored region. Blue and green lines represent additive effects in 12∶12 and 16∶8 respectively. (0.30 MB PDF) [file pgen.1001100.s004.pdf]

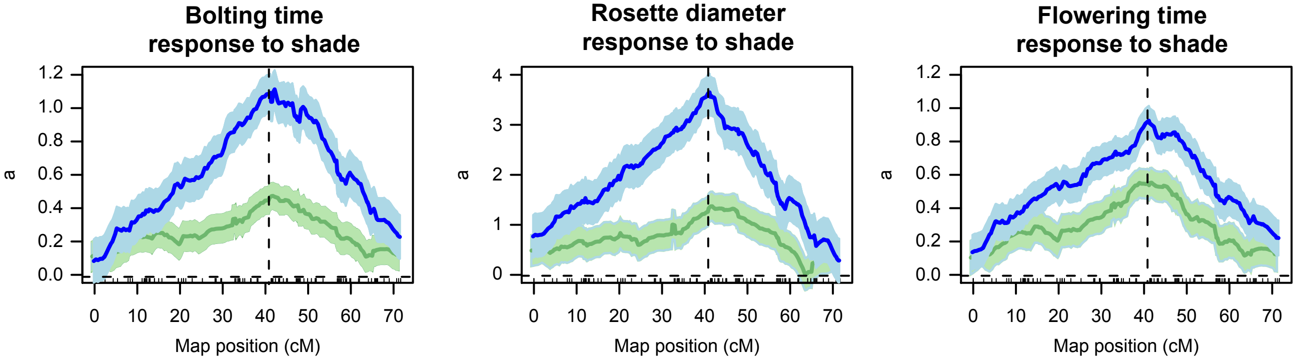

Bolting time
response to shade
Rosette diameter
response to shade
Flowering time
response to shade
a
1.2
1.0
0.8
0.6
0.4
0.2
0.0
4
3
2
1
0
0
10
20
30
40
50
60
70
Map position (cM)
